# TAO1 kinase maintains chromosomal stability by facilitating proper congression of chromosomes

**DOI:** 10.1098/rsob.130108

**Published:** 2014-06-04

**Authors:** Roshan L. Shrestha, Naoka Tamura, Anna Fries, Nicolas Levin, Joanna Clark, Viji M. Draviam

**Affiliations:** Department of Genetics, University of Cambridge, Cambridge CB2 3EH, UK

**Keywords:** mitosis, microtubule, kinetochore

## Abstract

Chromosomal instability can arise from defects in chromosome–microtubule attachment. Using a variety of drug treatments, we show that TAO1 kinase is required for ensuring the normal congression of chromosomes. Depletion of TAO1 reduces the density of growing interphase and mitotic microtubules in human cells, showing TAO1's role in controlling microtubule dynamics. We demonstrate the aneugenic nature of chromosome–microtubule attachment defects in TAO1-depleted cells using an error-correction assay. Our model further strengthens the emerging paradigm that microtubule regulatory pathways are important for resolving erroneous kinetochore–microtubule attachments and maintaining the integrity of the genome, regardless of the spindle checkpoint status.

## 2. Introduction

Microtubules attach to chromosomes via specialized multi-protein structures called kinetochores. Errors in kinetochore–microtubule attachment can result in chromosome segregation errors. Identifying how kinetochore–microtubule attachment is achieved is important to understand how cells prevent chromosome mis-segregation.

To prevent chromosome segregation errors, one sister of a kinetochore pair should attach to microtubules emanating from one spindle pole, and the other sister kinetochore should attach to microtubules from the opposing spindle pole in a configuration called ‘biorientation’. Distinct from the need to biorient, chromosomes face another geometrical problem: chromosomes predominantly are captured along the lateral walls of microtubules and then this lateral attachment should be converted to the ends of microtubules [1,2]. We recently showed that converting chromosome–microtubule attachments from an immature ‘lateral’ state to a mature ‘end-on’ state requires the end-on conversion process that is dependent on the microtubule destabilizer MCAK [3]. How mature kinetochore–microtubule attachments are ensured and biorientation is achieved are exciting and complex problems that are just beginning to be understood (reviewed in [2,4,5]).

TAO1/PSK2/MARKK/hKFC-B (hereafter called TAO1) is an upstream activator of MARK (a Par1 homologue), which phosphorylates microtubule-associated proteins and causes their detachment from microtubules leading to microtubule disassembly in interphase [6,7]. TAO kinases in the fly and mammals are well known to regulate interphase microtubule stability in response to actin signals (reviewed in [8]). It is not known whether TAO1's role in microtubule regulation is restricted to interphase or maintained during mitosis as well.

During mitosis, TAO1 kinase is active and is required for spindle orientation and cell rounding [9]. Whether TAO1 is required for chromosome segregation and congression has been less clear. In a functional genomic screen using RNAi, TAO1 was identified as a spindle checkpoint protein essential for the accurate segregation and proper congression of chromosomes [10]. Follow on studies excluded a role for TAO1 in the spindle checkpoint [11,12], but did not address TAO1's role in chromosome congression [10].

Here, we report new roles for TAO1 in maintaining mitotic microtubule dynamics, establishing proper chromosome–microtubule attachment and ensuring the accurate segregation of chromosomes. Our study confirms the previously described role for TAO1 in chromosome congression [10] and interphase microtubule stability [6,13]. By combining drug treatments with TAO1 depletions, we clarify TAO1's role in the spindle assembly checkpoint and establish the role of TAO1 in preventing errors in chromosome congression and segregation.

## 3. Results

### 3.1. Depletion of TAO1 causes chromosome congression defects

**Figure 1.**
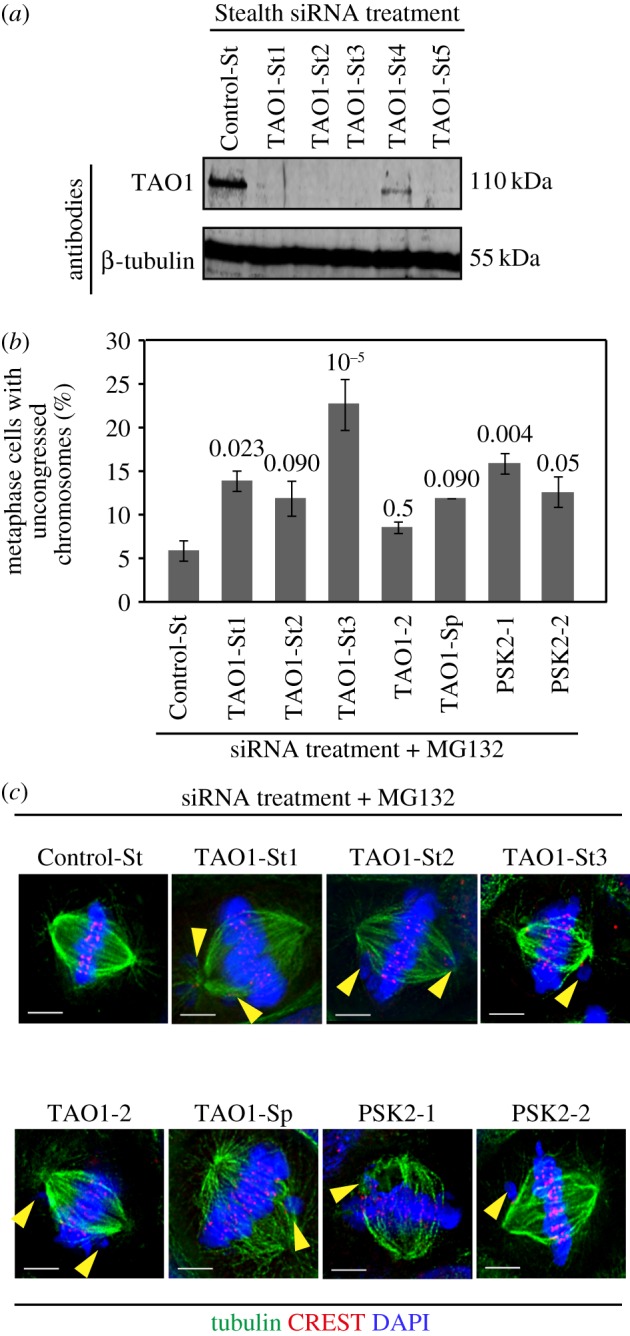
TAO1-depleted cells display chromosome congression defects. (*a*) Evaluation of TAO1 depletion extent using Stealth siRNA oligos. Immunoblots of lysates of HeLa cells transfected with the indicated Stealth siRNA oligos for 72 h and then probed with anti-TAO1 or anti-β-tubulin antibodies as indicated. β-Tubulin was used as a loading control. (*b*) Graph showing percentage of mitotic cells displaying uncongressed chromosomes in cells treated with siRNA as indicated and then arrested in metaphase for 90 min using MG132 prior to immunostaining using α-tubulin antibody and CREST antisera and staining with a DNA dye, DAPI. A total of *n* < 150 cells from three experimental repeats shown in electronic supplementary material, figure S1. Average and s.e.m. values (error bars) were calculated from three independent repeats (two shown in electronic supplementary material, figure S1). *p*-Values calculated using the proportion test are indicated above the bars. (*c*) Images of metaphase cells, from cultures treated as in (*b*), showing uncongressed chromosomes (marked with yellow arrowheads). Scale bars, 5 μm.

To circumvent siRNA-mediated off-target effects, we used the Stealth (St) RNAi system that eliminates sense strand off-target effects and screened for St-siRNA oligos that reproducibly deplete TAO1. Four out of five St-siRNA oligos against TAO1 (St1, St2, St3 and St5) showed approximately 90% depletion of TAO1, and three out of five St-siRNA oligos reproducibly depleted TAO1 (St1, St2 and St3) as quantified using fluorescent immunoblots (figure 1*a*; electronic supplementary material, figure S1 and data not shown). We first analysed chromosome congression efficiency in cells transfected with the three St-siRNA oligos (St1, St2 and St3) and four other published non-Stealth siRNA oligo sets against TAO1 (PSK2-1 and PSK2-2 [9], TAO1-Sp [11] and TAO1-2 [10]). For studying congression efficiency, we arrested cells at the metaphase–anaphase transition using MG132 (a proteasome inhibitor) and counted metaphase cells that presented uncongressed kinetochores. MG132-treated cells were immunostained with antibodies against α-tubulin (microtubule marker), CREST antisera (kinetochore marker) and a DNA dye, DAPI. TAO1 depletion using all seven siRNA oligos caused at least a twofold increase in cells with uncongressed kinetochores, compared to control depletions (figure 1*b*; electronic supplementary material, figure S1). One to three uncongressed kinetochore pairs were found adjacent to congressed kinetochores along the spindle walls in TAO1-depleted cells treated with MG132 (figure 1*c*). These findings confirm the role of TAO1 in congressing chromosomes, consistent with previous findings [10].

**Figure 2.**
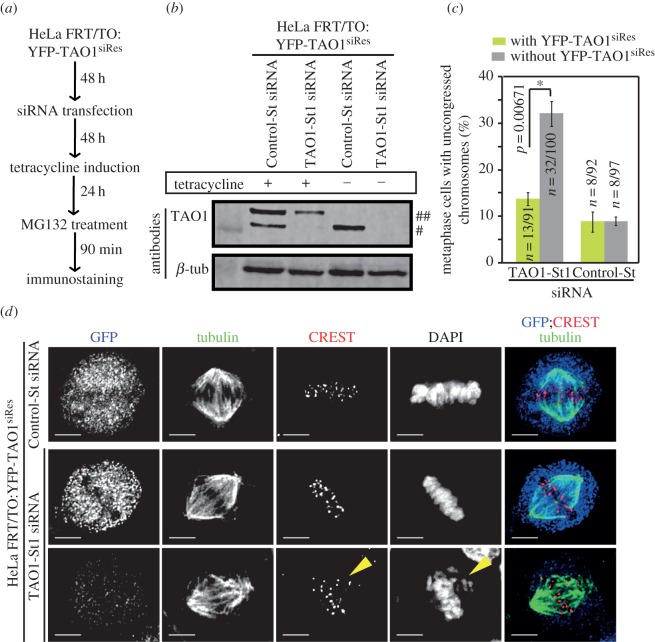
Rescue of TAO1 depletion-induced chromosome congression defects. (*a*) Schematic showing drug treatment regime for analysing kinetochore–microtubule attachment status. HeLa FRT/TO:YFP-TAO1^siRes^ cell line was treated with siRNA for 72 h, tetracycline-induced for 24 h and exposed to MG132 for 90 min prior to immunostaining cells with antibodies against GFP (for YFP) and tubulin, CREST antisera (centromeric marker) and DAPI (to stain for DNA). (*b*) Immunoblot of lysates of HeLa FRT/TO:YFP-TAO1^siRes^ cell line showing tetracycline-inducible expression of siRNA-resistant wild-type TAO1 (YFP-TAO1^siRes^) (marked as ##) in TAO1-St1 or Control-St siRNA-treated cells. In Control-St siRNA-treated cells, endogenous TAO1 can be visualized (marked as #). β-Tubulin (β-tub) was used as loading control. (*c*) Graph showing chromosome congression efficiency in TAO1-St1 or Control-St siRNA transfected and MG132-treated HeLa FRT/TO:YFP-TAO1^siRes^ cell line with or without YFP-TAO1^siRes^ expression. *n* = number of cells from three independent experiments. *p*-Values representing significance level were derived using proportion test. Asterisk (*) indicates significant difference. (*d*) Deconvolved immunoflourescence images of Control-St or TAO1-St1 siRNA transfected HeLa FRT/TO:YFP-TAO1^siRes^ cells with and without YFP-TAO1^siRes^ expression. Cells were transfected with siRNA as indicated and tetracycline-induced as shown in (*a*). Scale bar, 5 μm. Yellow arrowheads mark uncongressed chromosomes.

We next chose TAO1-St1 siRNA for our rescue studies since the nature of congression defects was reproducible across experimental repeats. To test whether TAO1-St1 siRNA treatment-induced defect in chromosome congression is specific to TAO1 depletion, we generated a cell line that conditionally expressed an siRNA-resistant form of TAO1 fused to YFP (YFP-TAO1^siRes^) in a tetracycline-inducible manner (HeLa FRT/TO:YFP-TAO1^siRes^) (figure 2*a*,*b*). This cell line when treated with tetracycline for 24 h resulted in a mixture of YFP-TAO1 expressing (green) and non-expressing (non-green) cells. The non-green cells served as internal negative controls. To evaluate chromosome congression efficiency, we treated cells with MG132 and then immunostained with antibodies against GFP (for probing YFP) and α-tubulin, CREST antisera (centromeric marker) and stained with DAPI. Control-St siRNA-treated cells predominantly displayed congressed chromosomes in metaphase, as expected (figure 2*c*). TAO1 siRNA-treated cells that were negative for YFP-TAO1 expression (non-green cells) displayed few uncongressed chromosomes that were away from the metaphase plate (figure 2*c*,*d*), consistent with figure 1*b*. By contrast, TAO1 siRNA-treated cells that were positive for YFP-TAO1 expression displayed fully congressed chromosomes, indicating successful rescue of TAO1 depletion induced chromosome congression defect. These findings demonstrate that the congression defect observed in TAO1-St1 siRNA-depleted cells is specifically due to TAO1 depletion.

### 3.2. TAO1 depletion reduces the growing fraction of microtubules during mitosis

**Figure 3.**
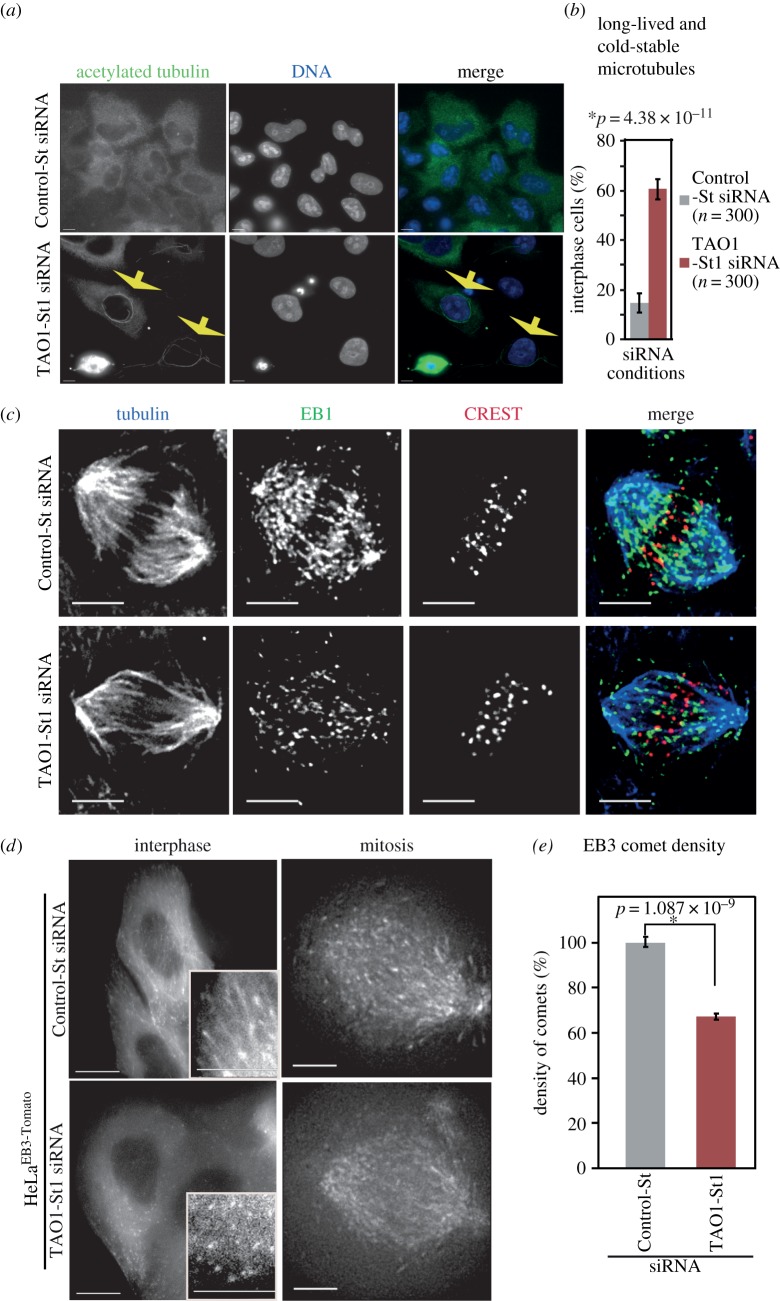
TAO1 is required to maintain dynamic microtubules during mitosis. (*a*) Immunofluorescence microscopy images showing the presence of ‘long-lived and cold-stable’ microtubules in TAO1-depleted cells (yellow arrows). HeLa cells were transfected with the indicated siRNA, cold treated for 20 min and immunostained with acetylated tubulin antibodies (green) and DAPI for DNA staining (blue). Scale bar, 5 μm. (*b*) Graph showing percentage of interphase cells displaying long-lived and cold-stable microtubules following siRNA and cold treatment as described in (*a*). *n* = number of cells from three independent experiments. (*c*) Deconvolved immunofluorescence images illustrating the reduction in EB1 comets following TAO1 siRNA treatment. HeLa cells were transfected with the indicated siRNA and immunostained with antibodies against α-tubulin (blue), EB1 (green) and CREST antisera (red). Scale bar, 5 μm. (*d*) Still images from time-lapse movies illustrating the reduction in comet density in HeLa^EB3-Tomato^ cells following TAO1-St1 or Control-St siRNA treatment. HeLa^EB3-Tomato^ cells were treated with siRNA as indicated and imaged during interphase or mitosis as indicated. Scale bar, 20 μm (interphase) and 5 μm (mitosis) images; inset 5 μm. (*e*) Graph of normalized average density of EB3 comets in HeLa^EB3-Tomato^ interphase cells counted as described in methods. *n* = 6 cells for each condition. Error bars are s.e.m. values across three experimental repeats. *p*-Values representing significance level were derived using the proportion test. Asterisks (*) indicate significant difference.

While TAO1 depletion causes an increase in interphase microtubule growth in *Drosophila* cells, TAO1 overexpression causes a loss of interphase microtubule cytoskeleton in human cells, supporting TAO1's role as a negative regulator of microtubule growth [6,13]. Whether TAO1 regulates microtubule lifetime during interphase and, importantly, whether TAO1 controls microtubule dynamics during mitosis has not been studied so far in any model organism. We first assessed for long-lived and cold-stable interphase microtubules by exposing cells to cold, a treatment that depolymerizes dynamic microtubules. Immunostaining of cells using an antibody against acetylated tubulin, a marker of long-lived microtubules, revealed that cold-stable and long-lived (acetylated) microtubules are increased following TAO1 depletion in HeLa cells (figure 3*a*,*b*). Cold-stable acetylated microtubule signals were often found in the perinuclear area of TAO1-depleted cells (figure 3*a*). These results show that TAO1 depletion leads to an increase in microtubule stability in HeLa cells, confirming previous reports on TAO1 function in *Drosophila* cells [13].

To confirm the specificity of TAO1 depletion inducing long-lived and cold-stable microtubules, we performed siRNA rescue experiments by expressing an siRNA-resistant form of YFP-TAO1 (YFP-TAO1^siRes^). Immunostaining cells using antibodies against acetylated tubulin and GFP (as YFP marker) showed that cold-stable acetylated microtubules are less abundant in TAO1 siRNA-treated cells expressing YFP-TAO1^siRes^ (electronic supplementary material, figure S2*a* and S2*b*), compared with TAO1 siRNA-treated cells that were not expressing YFP-TAO1^siRes^. As expected, control siRNA-treated cells did not display cold-stable acetylated microtubules (electronic supplementary material, figure S2*a* and S2*b*). These results confirm the specificity of TAO1 depletion inducing cold-stable and long-lived microtubules. We conclude that TAO1 is required for proper destabilization of interphase microtubules.

To measure the extent to which microtubule dynamics during mitosis is perturbed following the depletion of TAO1, we used two approaches: first, we co-immunostained HeLa cells with antibodies against EB1, a plus-end marker, and β-tubulin. Second, we quantified the fraction of dynamically growing microtubules in live cells expressing EB3-Tomato (HeLa^EB3-Tomato^). EB1 and EB3 specifically mark growing microtubule-ends and hence can be used as a measure of dynamic microtubules [14]. An obvious reduction in EB3 and EB1 comet density was observed in TAO1-St1 siRNA-treated cells, compared to Control-St siRNA-treated cells, during both interphase and mitosis (figure 3*c*–*e*; electronic supplementary material, figure S2*c* and S2*d*). A similar reduction in EB1 comet density was observed in cells treated with TAO1-St3 siRNA as well (electronic supplementary material, figure S3). These findings indicate a reduction in the growing fraction of microtubules following TAO1 depletion. Importantly, the overall astral microtubule length was not reduced in monopolar spindles of TAO1-depleted cells, compared with controls (electronic supplementary material, figure *S2e*). Also, bipolar spindle sizes were comparable between TAO1 and control-depleted cells (electronic supplementary material, figure S2*f*). Thus, a reduction in EB comet density without any reduction in microtubule length strongly suggests a decrease in the overall dynamics of microtubules following TAO1 depletion. This is fully consistent with the cold-stable long-lived microtubules observed in TAO1-depleted cells (figure 3*a*,*b*). In conclusion, TAO1 kinase controls the dynamic fraction of interphase and mitotic microtubule-ends. Thus, TAO1's role in microtubule regulation is not restricted to interphase but is maintained during mitosis.

### 3.3. TAO1 depletion promotes the incidence of mis-segregating lagging chromosomes

**Figure 4.**
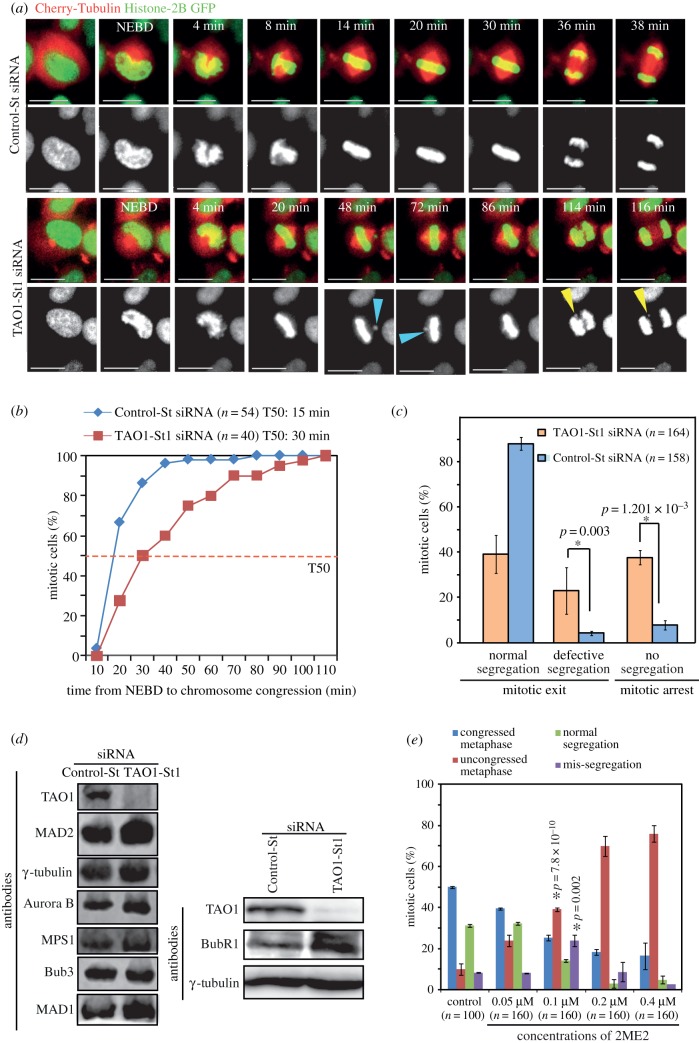
TAO1-depleted cells display mis-segregating lagging chromosomes. (*a*–*c*) Time-lapse analysis of HeLa^His2B-GFP;CherryTub^ cells transfected with TAO1-St1 or Control-St siRNA. (*a*) Successive frames of fluorescent images from representative live-cell movies of HeLa^H2BGFP;CherryTub^ cells treated with siRNA as indicated. Images were acquired once every 2 min. Uncongressed chromosomes are marked with blue arrowheads and mis-segregating lagging chromosomes are marked with yellow arrowheads. Scale bar, 20 μm. (*b*) Cumulative distribution graph showing the time consumed from NEBD to congression of chromosomes (alignment of last chromosome prior to anaphase) in cells treated with TAO1-St1 or Control-St siRNA. T50 represents chromosome congression time in 50% of mitotic cells. (*c*) Graph showing percentage of mitotic cells displaying normal, defective or no segregation within the first 3 h of mitosis. (*d*) Immunoblots of lysates of cells treated with siRNA as indicated showing the levels of Mad2, BubR1, Mad1, MPS1, TAO1, Aurora-B, Bub3 or γ-tubulin. γ-Tubulin is used as a loading control. (*e*) Graph showing percentage of mitotic cells displaying normal or uncongressed metaphase chromosomes and normal or mis-segregated anaphase chromosomes following a 1 h treatment with varying concentrations of 2ME2, as indicated. *n* = number of cells from three independent experiments. Error bars are s.e.m. values across three experimental repeats. *p*-Values representing significance level were derived using the proportion test. Asterisks (*) indicate significant difference.

To investigate the long-term consequence of the congression defect observed in TAO1-depleted cells, we imaged cells once every 2 min to follow spindle and chromosome movements in TAO1-St1 siRNA-treated HeLa cells expressing Histone-2B GFP and mCherry-Tubulin (HeLa^H2BGFP;CherryTub^). Quantifying the time consumed from nuclear envelope breakdown (NEBD) to congression completion (alignment of last chromosome) showed a noticeable delay in congression following TAO1 depletion (figure 4*a,b*). In TAO1-depleted cells, but not control cells, one or two chromosomes frequently lost alignment from the metaphase plate (figure 4*a*). This further confirms the role of TAO1 in chromosome congression, and explains the uncongressed kinetochores found in the fixed-cell studies (figure 1*c*).

While the majority of TAO1-depleted cells displayed anaphase delay, some cells displayed mis-segregating chromosomes (figure 4*a,c*). Mis-segregating chromosomes in TAO1-depleted cells were lagging or unaligned, consistent with the nature of congression defect. TAO1 depletion doubled the extent of chromosome mis-segregation (defective segregation: TAO1-St1 siRNA: 22 ± 10%; control-St-siRNA 4 ± 1%; figure 4*c*). Using quantitative fluorescent immunoblots, we confirmed that the levels of checkpoint proteins Mad1, Mad2, MPS1, BubR1, Bub3 and Aurora B are not lowered in TAO1-depleted cells (figure 4*d*), and thus ruled out the possibility of mis-segregation arising from a reduction in the levels of checkpoint proteins. We conclude that TAO1 is required for ensuring the proper congression and accurate segregation of chromosomes.

### 3.4. Perturbing microtubule stability causes both congression and segregation defects

Because TAO1-depleted cells showed increased microtubule stability, we tested the extent to which increasing microtubule stability *per se* could promote chromosome congression and segregation defects. For this purpose, we exposed HeLa cells to varying concentrations of 2ME2, a subtle microtubule stabilizing agent [15,16], for an hour and then immunostained cells using antibodies against tubulin, CREST antisera and DAPI. Quantifying the percentage of mitotic cells that displayed normal or abnormal metaphase revealed a 2ME2 dose-dependent increase in metaphase cells with congression defects (figure 4*e*). Importantly, abnormal anaphase, with segregation defects, was observed only at 0.1 μM concentration of 2ME2, but not at higher or lower concentrations of 2ME2 (figure 4*e*). This clearly demonstrates that chromosome congression and segregation defects can be induced to different extents by subtly varying microtubule stability.

### 3.5. TAO1 siRNA treatment-induced anaphase delay is specific to TAO1 depletion

To confirm that the segregation defects and anaphase delay observed in TAO1-St1 siRNA-treated cells are specifically due to TAO1 depletion (figure 4*a*), we returned to the cell line HeLa FRT/TO:YFP-TAO1^siRes^ in which we could control the expression levels of siRNA-resistant TAO1. To assess mitosis progression in this cell line, we transfected Histone-DsRed expression vector and generated a HeLa FRT/TO:YFP-TAO1^siRes^; His-Red cell line. Cells expressing both His-Red and YFP-TAO1 were very few and so anaphase onset was measured as the first time point when anaphase cell elongation was observed. Time-lapse movies of cells treated with TAO1-St1 siRNA showed a delayed anaphase onset in non-expressing (non-green) cells but no anaphase delay in YFP-TAO1^siRes^ expressing (green) cells (electronic supplementary material, figure S3*a*,*b*). Cells treated with control siRNA showed no obvious difference in anaphase onset between YFP-TAO1^siRes^ expressing and non-expressing cells (electronic supplementary material, figure S3*a*,*b*). These data show that the delayed anaphase onset observed following TAO1 siRNA treatment is rescued by expressing an siRNA-resistant form of TAO1 kinase. We conclude that TAO1 siRNA treatment-induced anaphase delay is specific to TAO1 depletion.

### 3.6. TAO1 depletion accelerates checkpoint satisfaction in Taxol-treated cells

**Figure 5.**
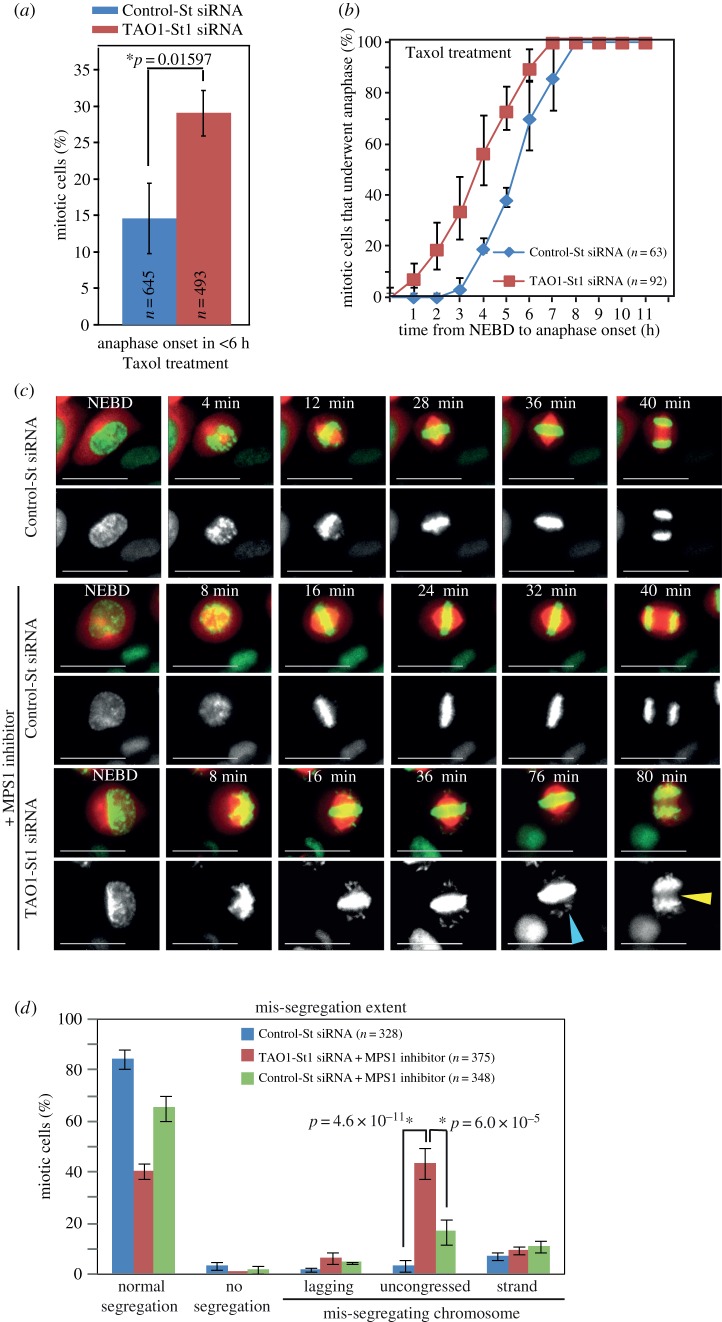
Taxol or MPS1 inhibitor treatments in TAO1-depleted cells. (*a*) Graph of percentage of cells that underwent anaphase, within 6 h of mitosis onset (NEBD), in HeLa^His2B-GFP;CherryTub^ cells transfected with TAO1-St1 or Control-St siRNA and exposed to Taxol during imaging. *n* = number of cells that entered mitosis during imaging from three independent experiments. (*b*) Cumulative distribution graph of NEBD to anaphase onset times in Control-St or TAO1-St1 siRNA-treated HeLa^His2B-GFP;CherryTub^ cells that exited mitosis in the presence of Taxol. *n* = number of cells that underwent anaphase from two different experiments. (*c*) Time-lapse images of cells treated with siRNA, as indicated, in the presence or the absence of 200 nM MPS1 inhibitor. Yellow arrowhead marks lagging chromosomes in anaphase and blue arrowhead marks unaligned chromosomes. Scale bar, 40 μm. (*d*) Graph showing mitotic outcome and mis-segregation severity by quantifying the percentage of cells that exhibited normal segregation, no segregation or mis-segregation. Mis-segregating cells were binned as those displaying lagging chromosomes, uncongressed chromosomes at metaphase–anaphase transition or lagging DNA strands as assessed from time-lapse movies as in (*c*). *n* = number of cells analysed from three independent experiments. Error bars are s.e.m. values across three experimental repeats. *p*-Values representing significance level were derived using the proportion test. Asterisks (*) indicate significant difference.

Time-lapse microscopy studies showed that TAO1-depleted cells display a slight anaphase delay suggesting a proficient checkpoint and in addition mis-segregating chromosomes suggesting a defective checkpoint (figure 4*a*). We tested whether TAO1-St1 siRNA-treated cells are capable of presenting a mitotic arrest in response to the microtubule poisons nocodazole and Taxol. HeLa^H2BGFP;CherryTub^ cells were treated with Control-St or TAO1-St1 siRNA and then exposed to nocodazole or Taxol at the beginning of time-lapse imaging. In the presence of 250 nM nocodazole, both Control-St and TAO1-St1 siRNA-treated cells arrested robustly in mitosis for a period of 18 h (Control-St siRNA: 232 cells; TAO1-St1 siRNA: 214 cells), showing a normal checkpoint. However, in the presence of 100 nM Taxol, cells treated with TAO1-St1 siRNA failed to arrest robustly in mitosis. In the presence of Taxol, while only 15% of Control-St siRNA-treated cells exited mitosis, nearly 30% of TAO1-St1 siRNA-treated cells exited mitosis within 6 h (Control-St siRNA: 645 cells; TAO1-St1 siRNA: 493 cells) (figure 5*a*). Importantly, analysis of anaphase onset times showed that TAO1-depleted cells initiated anaphase earlier than control-depleted cells in the presence of Taxol (figure 5*b*). This shows that Taxol treatment combined with TAO1 depletion can accelerate checkpoint satisfaction, consistent with microtubule stabilizing conditions promoting segregation errors (figure 4*e*) [17].

### 3.7. TAO1 depletion enhances defects induced by partial inhibition of MPS1 function

To further confirm TAO1's role in ensuring proper kinetochore–microtubule attachment, we used an alternative approach of exposing cells to a low concentration of MPS1 inhibitor, NMS-P715 [18]. At 200 nM concentration of MPS1 inhibitor treatment, more than 65% of Control-St siRNA-treated HeLa^H2BGFP;CherryTub^ cells underwent anaphase with normal chromosome segregation (figure 5*c,d*). By contrast, only 40% of TAO1-St1 siRNA-treated cells underwent anaphase with normal segregation (figure 5*c*). Importantly, in MPS1 inhibitor-treated cells the severity of chromosome mis-segregation was much higher following TAO1 depletion (figure 5*c*,*d*). Also, quantifying the kind of mis-segregation showed that disrupting both MPS1 and TAO1 function together causes an increase in the mis-segregation of uncongressed chromosomes scattered along the spindle walls (figure 5*c*,*d*). However, other kinds of mis-segregation such as lagging chromosomes and lagging DNA strands were comparable between TAO1- and control-depleted cells under MPS1 inhibitor treatment conditions. These findings show that TAO1 depletion in combination with partial inhibition of MPS1 induces severe mis-segregation of chromosomes, further confirming TAO1's role in ensuring proper chromosome–microtubule attachment.

### 3.8. TAO1 is required for resolving erroneous chromosome–microtubule attachments

**Figure 6.**
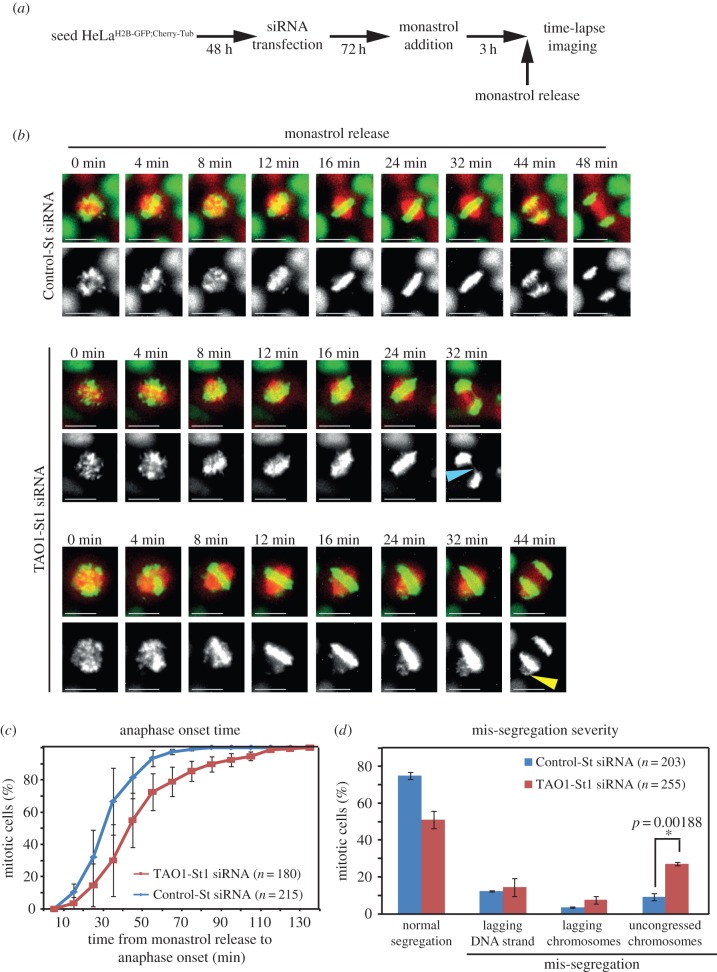
Correcting erroneous kinetochore–microtubule attachments requires TAO1. (*a*) Schematic of assay to measure error-correction efficiency showing time-points for monastrol treatment and release prior to time-lapse imaging of HeLa^His2B-GFP;CherryTub^ cells treated with either TAO1-St1 or Control-St siRNA. (*b*) Time-lapse images of cells treated with siRNA, as indicated, undergoing anaphase following a release from monastrol treatment. Blue arrowhead marks lagging DNA and yellow arrowhead marks unaligned and mis-segregating chromosomes. Scale bar, 20 μm. (*c*) Cumulative distribution graph of anaphase onset time in Control or TAO1-depleted cells following a release from monastrol treatment, accrued from time-lapse movies as in (*b*). (*d*) Graph of mis-segregation severity quantifying the percentage of cells that exhibited normal segregation or mis-segregation with lagging DNA strands or lagging chromosomes or uncongressed chromosomes, assessed from time-lapse movies as in (*b*). *n* = number of cells analysed from three independent experiments. Error bars are s.e.m. values across three experimental repeats. *p*-Values representing significance level were derived using the proportion test. Asterisk (*) indicates significant difference.

To quantify the extent to which TAO1 is critical for preventing errors in chromosome–microtubule attachments and segregation, we used an assay developed to expose error-correction defects [19]. By transiently altering spindle geometry using monastrol, an Eg5 inhibitor, monopolar to bipolar spindle conversion can be synchronized and imaged in hundreds of mitotic cells, which allows careful quantitative analysis of error-correction defects and mis-segregation severity. Forty-five minutes after monastrol wash-out, a majority of control cells assembled bipolar spindles, congressed chromosomes and initiated chromosome segregation, as expected. By contrast, TAO1-depleted cells showed a delay in anaphase onset with persistent unaligned chromosomes (figure 6*b*,*c*). The prolonged presence of unaligned chromosomes following TAO1 depletion is indicative of a failure in resolving erroneous kinetochore–microtubule attachments.

Quantifying mis-segregation extent showed that a majority of control-depleted cells underwent normal segregation and a small minority exhibited mis-segregation (figure 6*d*), consistent with previous findings [20]. By contrast, TAO1-depleted cells showed an increase in the extent of chromosome mis-segregation compared to control-depleted cells following a monastrol wash-out treatment (figure 6*d*). Particularly, the nature of mis-segregation was different between TAO1- and control-depleted cells. While unaligned mis-segregating chromosomes scattered along the spindle walls were significantly increased in TAO1 compared with control-depleted cells, lagging DNA strands were comparable in both TAO1-depleted cells and control cells. An increased incidence of chromosome mis-segregation shows a role for TAO1 in resolving erroneous kinetochore–microtubule attachments.

## 4. Discussion

Using several drug treatments, monastrol-release (error-correction) assay, partial MPS1 inhibition and Taxol treatment, we confirm the role of TAO1 in ensuring proper congression and accurate segregation of chromosomes. We uncover a role for TAO1 in maintaining the growing fraction of microtubules in both mitosis and interphase in human cells. We propose that the microtubule regulatory function of TAO1 is important for resolving erroneous chromosome–microtubule attachment and in turn preventing congression and segregation defects. Consistent with this model, we find that microtubule stabilization induced by the drug 2ME2 can also cause congression and segregation defects, in a dose-dependent manner. Our data strongly support the emerging paradigm that microtubule destabilizing pathways are important to prevent chromosomal instability, regardless of the spindle checkpoint status.

Understanding a kinase's role using a loss of function approach such as RNAi is often made difficult by partial protein depletions, as previously discussed for checkpoint kinases, Bub1 and Aurora B [21,22]. To circumvent this difficulty at least in part, we have included (i) multiple siRNA oligos to assess phenotypic consistency, (ii) rescue studies with siRNA oligo-resistant TAO1 cDNAs to confirm RNAi specificity and (iii) partial depletion studies to cross-validate the loss of protein function. siRNA treatment conditions that failed to fully deplete TAO1 failed to perturb chromosome congression, demonstrating that an efficient depletion of TAO1 kinase is essential to show TAO1's mitotic role.

This study confirms TAO1's role in congression and clarifies TAO1's role in segregation. We use Taxol treatment, monastrol wash-out regimen and partial MPS1 inhibitor treatment to fully expose the role for TAO1 in preventing errors in chromosome congression and segregation. The extent of segregation errors observed in TAO1-depleted cells is not as severe as the one described in Draviam *et al.* [10], and this is consistent with the nature of congression defects and anaphase onset delay observed following TAO1 depletion.

**Figure 7.**
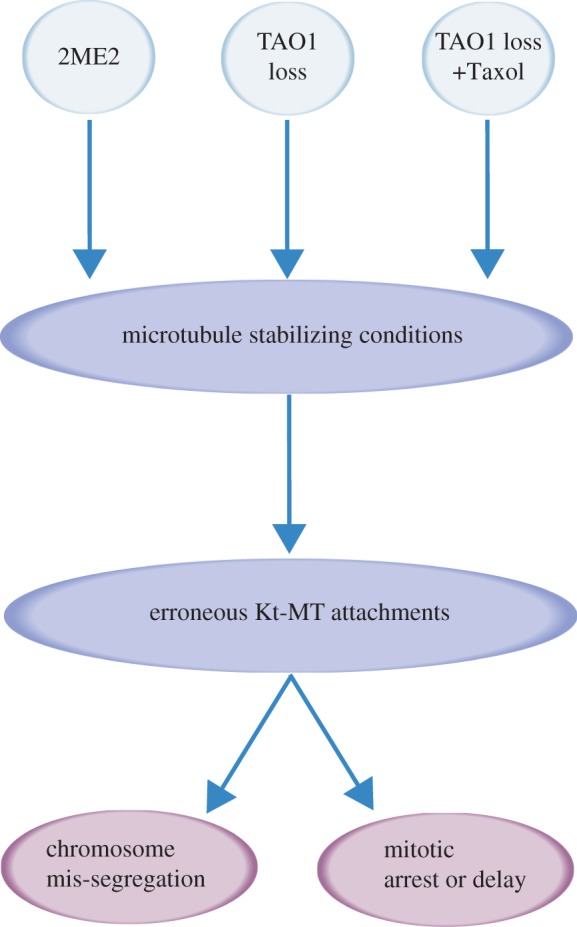
Model of TAO1 depletion-induced mis-segregation arising from defective microtubule stability. Microtubule stabilizing conditions (2ME2 treatment, TAO1 depletion, TAO1 depletion combined with Taxol treatment) all promote errors in kinetochore–microtubule (Kt-MT) attachments. Regardless of the spindle checkpoint status, microtubule stabilizing conditions ultimately promote two distinct mitotic outcomes: mitotic arrest or delay and mitotic exit with mis-segregation.

Microtubule stabilizing conditions such as 2ME2 treatment (this study), Taxol treatment [17] and MCAK depletion [23] present strong evidence for microtubule stabilization-induced errors in chromosome–microtubule attachment and chromosome segregation, regardless of checkpoint status. Increased cold-stable acetylated tubulin signals and decreased EB comet density following TAO1 depletion confirm its previously reported role in regulating microtubules [6]. We therefore speculate that the underpinning cause for congression and segregation errors following the depletion of TAO1 is enhanced microtubule stability (figure 7). Changes to kinetochore–microtubule attachment stability and mitosis length are also reported in cells lacking proteins of the hippo tumor suppressor pathway: MST1, MST2, NDR1 and RASSF1A [24–27]. With the TAO family of kinases being able to phosphorylate and activate the hippo homologues MST1 and MST2 kinases in flies and humans [28,29], it will be useful to learn about how the kinases of oncogenic signalling pathways work together to ensure proper kinetochore–microtubule attachments and prevent chromosomal instability.

## 5. Material and methods

### 5.1. Cell culture, transfection and synchronization

HeLa cells were cultured in Dulbecco's modified Eagle's Media (DMEM) supplemented with 10% fetal calf serum (FCS) and antibiotics, penicillin and streptomycin). For inhibition studies, cells were treated with 10 µM monastrol (1305, Tocris) for Eg5 inhibition for 3 h or 200 nM NMS-P715 (475949-5MG, Merck) for MPS1 inhibition prior to filming or fixation. 2ME2 (Sigma) was added directly to DMEM containing FCS (Invitrogen). Cells were transfected with siRNAs as described [30]. All siRNA oligos used against TAO1 are listed in the electronic supplementary material. For Control-St siRNA treatment, negative control (12935-300, Invitrogen) was used. All siRNA transfections were performed using Oligofectamine (Life Technologies) according to the manufacturer's instructions. For tetracycline induction, cells were treated with 1 ng ml^–1^ tetracycline (Sigma).

### 5.2. Cell line generation

HeLa^H2BGFP;CherryTub^ [16] and HeLa^EB3-Tomato^ [31] were sorted using FACS to enrich for cells expressing uniform amount of fluorescent reporter protein expression. Stable isogenic HeLa FRT/TO:YFP-TAO1^siRes^ cell line was generated by transfecting pcDNA5/FRT/TO:YFP-TAO1^siRes^ along with Flp-recombinase expression vector pOG44 (1 : 9 ratio) into a Flp-In T-Rex HeLa cell line according to the manufacturer's instructions (Life Technologies). Forty-eight hours after transfection, cells were selected using hygromycin (Sigma) and positive colonies were picked for expansion. The best clone was selected after confirming that more than 80% of cells express YFP-TAO1, selectively after induction with tetracycline. To generate the HeLa FRT/TO:YFP-TAO1^siRes^; His-Red cell line, Histone 2B-DsRed pcDNA2.1 [30] was transfected using electroporation (Neon Transfection System) into the HeLa FRT/TO:YFP-TAO1^siRes^ cell line. Positive cells were enriched using FACSorting.

### 5.3. Live-cell time-lapse imaging and analysis

Cells were transfected with siRNA oligos or plasmids 24 h after seeding in LabTek 0.15 mm thickness glass dishes (Nunc). Twenty-four hours after plasmid transfection, cells were used for analysis. Seventy-two hours after siRNA transfection, cells were transferred to Leibovitz's L15 medium (Invitrogen) and filmed at 37°C. For long-term live-cell imaging, exposures of 0.1 s and at least three *Z*-planes were acquired for 4 h using a 40× NA 0.75 objective on an Applied Precision Deltavision RT microscope equipped with a Mercury 100 W lamp, GFP-long pass filter (Chroma, Rockingham, VT, USA), phase-contrast filters and Coolsnap HQ camera. For short-term high-resolution live-cell studies, exposures of 0.04–0.2 s for 2–5 min and at least three *Z*-planes were acquired using a 100× NA 1.2 objective on the microscope described above using an EMCCD camera. A frame rate of 0.2–0.9 s was used for EB comet studies and 2–4 min was used for chromosome tracking studies. The timing of anaphase onset from NEBD was determined as previously described [30] in all figures except electronic supplementary material, figure S4.

### 5.4. Long-lived and cold-stable microtubule assessment, microtubule length measurement

For analysing cold stability, cells were exposed to cold for 20 min. Cells were seeded on coverslips grown on 12-well dishes. Seventy-two hours post-siRNA transfection, the 12-well dish was placed on a bed of ice for 20 min and then fixed using ice-cold methanol prior to immunostaining using antibodies as indicated. Immunostained cells were assessed for the presence of acetylated and cold-stable microtubules. At least 100 cells from four distinct positions of the coverslip were considered for the graphs.

Microtubule length measurements are measured as described in detail in Corrigan *et al.* [16]. Briefly, monastrol-treated cells were immunostained with antibodies against α-tubulin, CREST antisera and DAPI. Spindle pole to microtubule tip distance was measured using softWoRx.

### 5.5. Statistical analysis

*P*-values were obtained using the proportion test or unpaired Student's *t*-test.

### 5.6. EB3 comet density measurement

Two non-overlapping 5 μm^2^ areas from a single *Z*-plane image of a cell in a time-lapse movie were used to manually count the number of comets. Using these data, the average number of comets per square micrometre per cell was computed. Comet density was then normalized against values in Control-St siRNA-treated cells and *p*-values were determined.

### 5.7. Error-correction efficiency assay

HeLa^H2BGFP;CherryTub^ cells were treated with siRNA oligos for 72 h. Three hours prior to filming, cells were treated with DMEM media containing 10 μM monastrol. To monitor monopolar to bipolar conversion and anaphase onset, the cells were washed three times with PBS and then released into fresh Leivobitz media (L15) and imaged using a 40× objective once every 2–4 min, as indicated.

### 5.8. TAO1_1.2_VMD monoclonal antibody generation

His tagged TAO1 (722–1001a.a; Q7L7X3) expressed in *Escherichia coli* was purified using His Select Nickel affinity Gel (Sigma-P6611). Purified protein was then sent to Moravian Biotech for generating mouse monoclonal antibody against TAO1. Different clones of hybridoma cells were screened to obtain a suitable clone that produced specific antibody against full-length TAO1. Hybridoma cells were grown in DMEM media supplemented with 10% FCS, penicillin and streptomycin (Invitrogen) according to the manufacturer's instructions. Once cells reached 90% confluency, cells were centrifuged at 1200 r.p.m. for 3 min. The supernatants (media) containing mouse monoclonal antibody were separated and stored at 4°C. For immunoblotting, supernatant (diluted in 1 : 2 ratio with PBS containing 0.1% Tween and 5% dry milk) of hybridoma cells was incubated overnight at 4°C, prior to washing and probing with secondary anti-mouse fluorescent antibody.

### 5.9. Quantification of protein depletion

Fluorescent immunoblots were imaged using LI-COR and band intensities were quantified using LI-COR Odyssey software. Briefly, boxes were drawn as close to band intensities as possible. Intensity/pixel values were calculated for the boxed area by subtracting the background intensity from total intensity. Ratio of intensity/pixel values corresponding to TAO1 and γ-tubulin bands were computed and plotted as percentages by normalizing against values obtained from Control-St siRNA condition.

### 5.10. Immunostaining and immunoblotting

For immunofluorescence, cells were grown on coverslips and fixed after washing once in PBS with ice-cold methanol for 1 min, followed by blocking with 1% BSA in PBS/0.1% Tween for 30 min at room temperature. Coverslips were incubated in primary antibodies for 1 h, washed thrice in PBS with 0.1% Tween and incubated with secondary antibodies for 45 min. Cells were then stained with DAPI and mounted on slides using Prolong antifade mounting media (Invitrogen). Mouse anti EB1 (BD- 610534) and rat anti-α-tubulin (Abcam ab6160) were used at 1 : 500 dilution. Mouse anti-tubulin (T4026) and mouse anti-acetylated tubulin (T6793-2ML) both from Sigma were used at 1 : 200 dilution. Mouse anti-GFP (Roche-11814360001) was used at 1 : 1000 dilution. Human ANA-Centromere CREST Autoantibody (Europa Bioscience FZ90C-CS1058) was used at 1 : 1500 dilution. Secondary antibodies used were Alexa Fluor 488, 594 and 647 (Invitrogen) and Dylight 488 anti-rat IgG (Abcam ab98420) at a dilution of 1 : 500. For immunoblotting, rabbit anti-TAO1 (A300-524A) and rabbit anti-Mad2 (A300-300A) antibodies, both from Bethyl Laboratories, were used at 1 : 300 dilutions. Rabbit anti- BubR1 (Bethyl Laboratories-A300-995A), rabbit anti-Aurora B (Abcam ab2254), rabbit anti-Bub3, mouse anti-MPS1 and rabbit anti-Mad1 antibodies [27] were used at 1 : 500 dilutions. Mouse anti-γ-tubulin antibody (Sigma) was used at 1 : 800 dilution.

### 5.11. Plasmids and siRNA transfection

The primers 5′-GCAGTGAATTCTATGCCATCAACTAACAGAG-3′ and 5′-GCATTGGATCCTTATGTATAAGACATGTGTGAC-3′ were used to amplify TAO1 and subclone it into EcoR1 and BamHI sites of pEYFPC1 vector (Clontech). The siRNA-resistant TAO1 construct (YFP-TAO1^siRes^) was generated by wobbling base-pairs corresponding to TAO1-St1 siRNA oligo by point mutagenesis using the following primers: 5'-CGTCGCTTCAAGAGGAGGATGTTGCTCGGACGTCACAACTTAGAGC-3’ and 5'-GCTCTAAGTTGTGACGTCCGAGCAACATCCTCCTCTTGAAGCGACG-3’. Pme1 site was introduced into pcDNA5 FRT/TO (Invitrogen) to allow the incorporation of YFP-TAO1^siRes^ at the Pme1 site using the primers 5′-ATTCAAGTTTAAACATGGTGAGCAAGGGCGAGGAG-3′ and 5′-ATTCAAGTTTAAACTTATGTATAAGACATGTGTG-3′.

HeLa cells were transfected with siRNAs as described [27]. siRNA oligos used and their sequences are listed in the electronic supplementary material, table S1. For Control-St siRNA, Negative control oligo (12935-300, Invitrogen) was used.

## Supplementary Material

Supplementary Figure 1

## Supplementary Material

Supplementary Figure 2

## Supplementary Material

Supplementary Figure 3

## Supplementary Material

Supplementary Figure 4
