# Supplementary figures and images for: TAO1 kinase maintains chromosomal stability by facilitating proper congression of chromosomes
Source: Open Biol. 2014 Jun 4;4(6):130108. doi: 10.1098/rsob.130108 (PMC4077056; doi:10.1098/rsob.130108)

Repeat 1

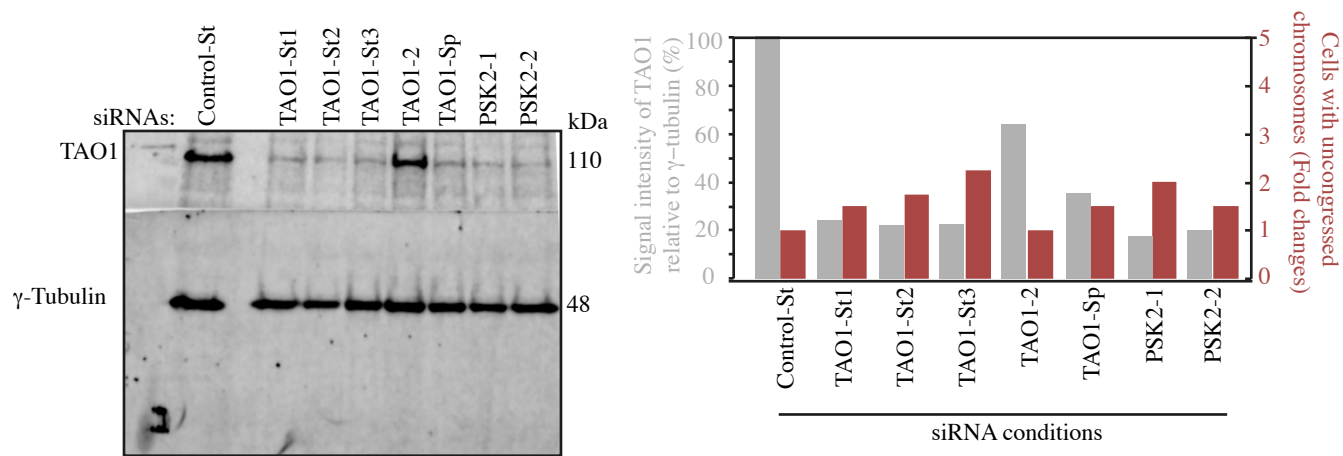

Repeat 2

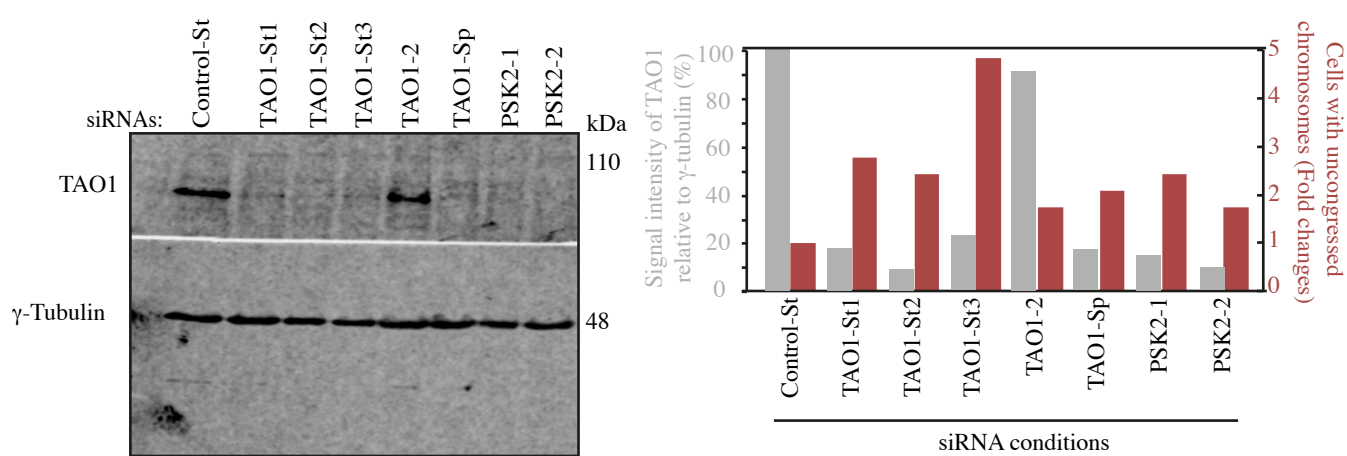

Supplement: Supplementary Figure 1 [file rsob130108supp1.pdf]

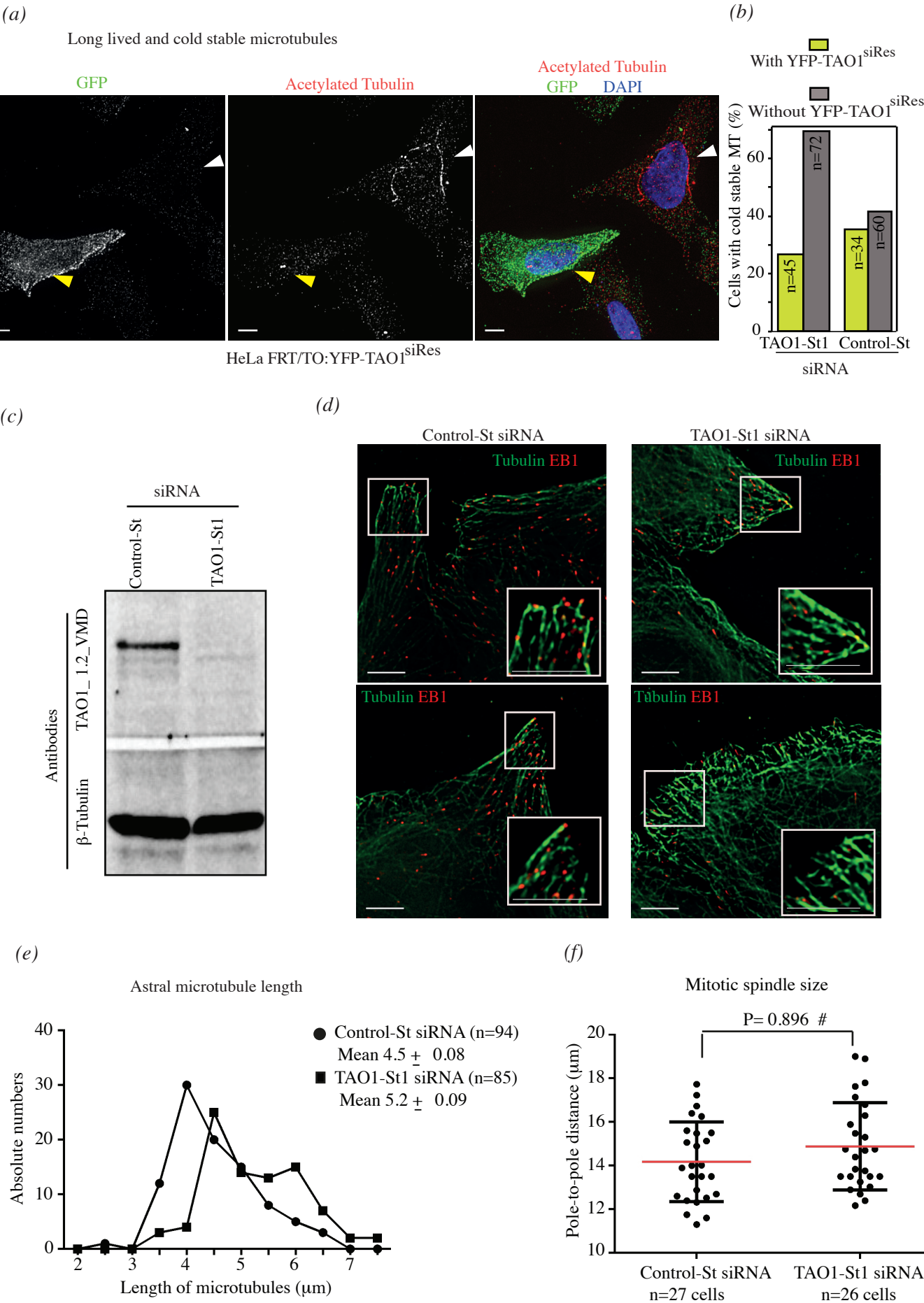

Supplement: Supplementary Figure 2 [file rsob130108supp2.pdf]

(a)

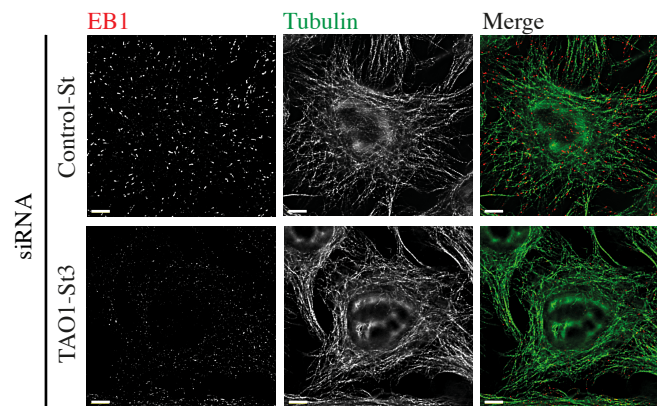

(b)

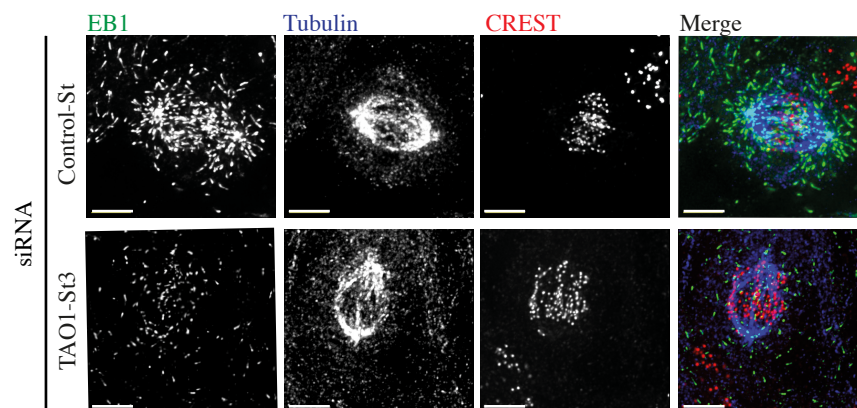

(c)

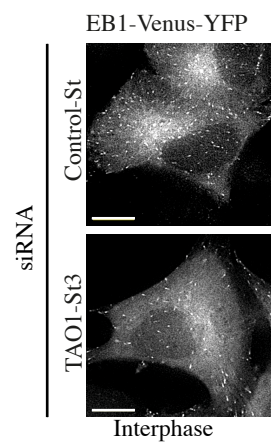

(d)

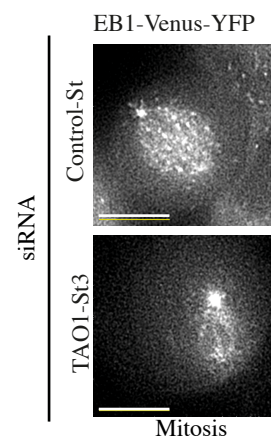

Supplement: Supplementary Figure 3 [file rsob130108supp3.pdf]

(a)

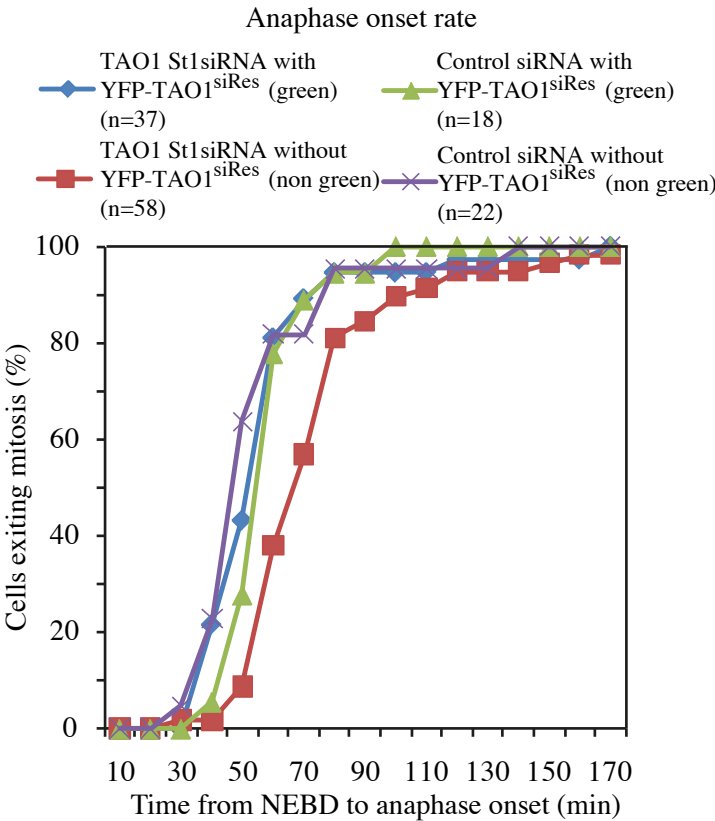

(b)

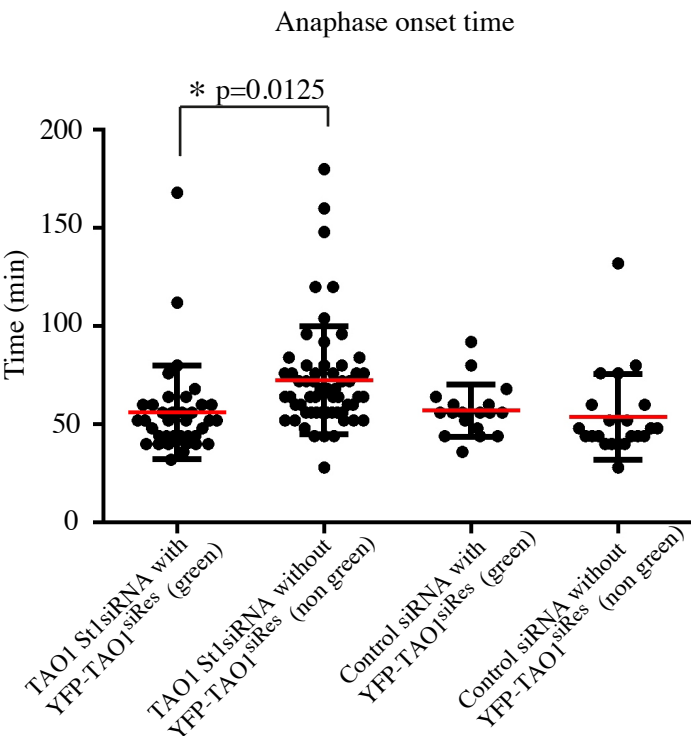

Supplement: Supplementary Figure 4 [file rsob130108supp4.pdf]
